# Focal Unspecific Bone Uptake on [^18^F]PSMA-1007 PET: Evaluation Analog PROMISE Criteria and Validation via PET/CT Follow-Up

**DOI:** 10.3390/diagnostics14202327

**Published:** 2024-10-18

**Authors:** Jonas-Alexander Benecke, Eduardo Calderón, Gerald Reischl, Andreas Brendlin, Igor Tsaur, Christian la Fougère, Jonas Vogel

**Affiliations:** 1 Nuclear Medicine and Clinical Molecular Imaging, Department of Radiology, University Hospital of Tuebingen, Otfried-Mueller-Strasse 14, 72076 Tuebingen, Germany; jonas-alexander.benecke@med.uni-tuebingen.de (J.-A.B.); eduardo.calderon-ochoa@med.uni-tuebingen.de (E.C.); jonas.vogel@med.uni-tuebingen.de (J.V.); 2 Cluster of Excellence iFIT (EXC 2180) “Image Guided and Functionally Instructed Tumor Therapies”, University of Tuebingen, 72076 Tuebingen, Germany; 3 Werner Siemens Imaging Center, Department of Preclinical Imaging and Radiopharmacy, Eberhard Karls University, 72076 Tuebingen, Germany; 4 Diagnostic and Interventional Radiology, Department of Radiology, University Hospital of Tuebingen, Hoppe-Seyler-Str. 3, 72076 Tuebingen, Germany; 5 Department of Urology, University Hospital of Tuebingen, Hoppe-Seyler-Str. 3, 72076 Tuebingen, Germany; 6 German Cancer Consortium (DKTK), Partner Site Tuebingen, 72076 Tuebingen, Germany

**Keywords:** prostate cancer, [^18^F]PSMA-1007, PET/CT, unspecific bone uptake, bone lesion, bone metastases

## Abstract

**Background:** Focal unspecific bone uptake (UBU) is common in [^18^F]PSMA-1007 PET/CT, yet its clinical significance remains unclear, causing uncertainty in treatment decisions. **Material and Methods:** We retrospectively analyzed 99 prostate cancer patients (age 69 ± 7) who underwent [^18^F]PSMA-1007 PET/CT scans (3 MBq/kg; uptake time 70 ± 14 min) for staging and follow-up (after 13.0 ± 7.2 months). Semiquantitative assessment using the miPSMA score, analogous to the PROMISE criteria, evaluated the prevalence of UBU and bone metastases. **Results:** In the initial PET/CT scan, 56 patients had 230 lesions classified as UBU. A total of 19 patients were found to have bone metastases and UBU, while 24 patients had no focal bone uptake. UBU distribution was as follows: ribs (50%), spine (30%), pelvis (15%), and other sites (5%). There were no significant differences in age, Gleason score, injected tracer dose, uptake time, SUV_peak_ of UBU, or SUV_mean_ in the spleen and parotid gland between patients with and without UBU. Follow-up showed stable miPSMA-score and CT appearance in 44/56 patients with UBU (79%), minor changes in 5/56 patients (8%), and new bone metastases in 7/56 patients (12%). Patient-specific analysis indicated at least one bone metastasis initially classified as UBU in 3/56 patients (5%) and new bone metastases in 4/56 patients (7%). In total, 4 of the 24 patients (17%) without initial focal uptake developed osseous metastases at follow-up. **Conclusions:** No significant differences were found between patients with or without UBU. Only a small portion of UBU (2%) evolved into metastases, a lower rate than the development of new osseous metastases, which appears to be independent of UBU.

## 1. Introduction

Prostate cancer represents the most common malignancy in men [1], resulting in a considerable proportion of clinical imaging examinations focusing on patients with this tumor entity. In this regard, multiple studies have validated the superior efficacy of positron emission tomography (PET) targeting the prostate-specific membrane antigen (PSMA), an transmembrane protein overexpressed on prostate cancer cells, over conventional cross-sectional imaging modalities [2,3,4,5].

In addition to the initially used gallium-68 labeled PSMA ligands, different PSMA-targeting tracers utilizing fluorine-18 are currently emerging, with [^18^F]PSMA-1007 being the primary tracer employed [6]. In ^68^Ga-PSMA ligands, chelators are used to bind the radiometal, whereas in [^18^F]PSMA-1007, no chelator complex is necessary as fluorine-18 is bound covalently to fluoronicotinic acid, which is introduced into the molecule together with additional hydrophilic groups without influencing the pharmacophore binding to PSMA [7]. [^18^F]PSMA-1007 exhibits superior biodistribution compared to [^68^Ga]Ga-PSMA-11, characterized by reduced bladder excretion and the consequent improvement of locoregional staging [6,8,9,10].

Furthermore, the physical properties of fluorine-18, notably its longer half-life and production in a cyclotron, contribute to improved availability and lower manufacturing costs [11,12,13]. Additionally, due to the lower positron energy of fluorine-18 (0.65 MeV vs. 1.92 MeV), the spatial resolution is improved in [^18^F]PSMA-1007 PET scans.

However, the use of [^18^F]PSMA-1007 has been associated with an elevated prevalence of focal unspecific bone uptake (UBU) in up to 72% of patients, which appears to be significantly more predominant than in other tracers [6,14]. Moreover, several studies have shown increases in the prevalence of UBU in digital PET scanners [15,16,17,18]. UBU is classified as focal osseous tracer uptake that does not meet the criteria of osseous metastases defined by PROMISE regarding lesion number, miPSMA score, and morphological appearance on computer tomography (CT). According to this definition, classification is performed as follows: focal bone uptake is considered a bone metastasis if a sclerotic or lytic CT lesion displays a peak standard uptake value (SUV_peak_) greater than the SUV_mean_ of the blood pool or if there is an equivocal CT lesion with an SUV_peak_ higher than the SUV_mean_ of the spleen. Without a CT correlate, a solitary focal uptake must show a higher uptake than the SUV_mean_ of the parotid gland to be classified as bone metastasis, and in the case of multifocal bone uptake, lesion uptake must be greater than the SUV_mean_ of the spleen. Lesions with benign CT morphology or non-prostate cancer-specific malignant lesions are considered bone metastases if the SUV_peak_ is > the SUV_mean_ of the parotid gland [19].

The cause of UBU, in particular the increased prevalence with the use of [^18^F]PSMA-1007, remains unclear [20,21]. The hypothesis that the use of an ^18^F-labeled radiotracer leads to the potential presence of free fluorine-18, which may constitute the pathophysiological correlate of bone accumulation, could not be substantiated [22]. Moreover, this explanation fails to account for variable prevalence among ^18^F-labeled radiotracers [15,23]. Current data also argue against a PSMA-mediated specific accumulation, which also does not explain the high variability between different PSMA-targeting radiotracers [15,20,21,24,25]. Of note, Vollnberg et al. demonstrated that PSMA-avid bone lesions biopsied under PSMA-PET/CT guidance did not show histological PSMA expression [24]. In addition, immunohistochemical investigations failed to demonstrate increased PSMA expression in normal bone marrow [26].

Because of this, underlying benign bone and bone marrow changes are considered the most likely cause of UBU [15,21], but the current data for such a clinically relevant interpretation are still rather limited, so further studies seem necessary. In various studies and case reports, the histological confirmation of UBU has revealed benign osseous changes such as Paget’s disease, hyperplastic bone marrow, and fibrous dysplasia [15,21,27,28]. However, due to their low incidence, these are certainly only occasionally the cause of UBU. Ninatti et al. support the hypothesis that underlying osteoporosis-related remodeling could be the cause of UBU and better correlates with the high prevalence of this finding [29]. The differing chemical structures and pharmacokinetic properties of different new tracers could also explain the increased occurrence of UBU with highly specific tracers such as [^18^F]PSMA-1007 [30].

On the other hand, intermodal correlation [20], follow-up imaging [15,31], and histopathological assessment [24] have shown that a proportion of UBU may represent early-stage osseous metastases. The absence of CT morphological correlates supports the high sensitivity of molecular diagnostics in detecting (pathological) metabolic processes before the onset of morphological changes. However, the prevalence of malignant findings varies widely in recent studies, ranging from 0% to 22.8% [21,31]. Therefore, the clinical significance of these findings remains elusive in clinical practice, contributing to increasing uncertainty in image analysis and potentially leading to the over- or undertreatment of patients.

Therefore, the aim of our study was not only to describe the presence and distribution pattern of UBU according to the PROMISE criteria but also to evaluate the incidence of malignant transformation of identified UBU in order to improve our understanding of their clinical significance for [^18^F]PSMA-1007, a tracer used worldwide and approved in different countries. Although histological confirmation represents the gold standard, it is neither ethically nor practically feasible in larger cohorts due to its invasiveness. Accordingly, we evaluated the incidence via follow-up imaging using the same imaging protocol, radiotracer, and PET/CT scanner.

## 2. Materials and Methods

### 2.1. Patient Cohort

In this study, we retrospectively analyzed patients who underwent [^18^F]PSMA-1007 PET/CT imaging at our institution between May 2018 and March 2021. Our local institutional ethics committee reviewed and approved this study (Project number: 064/2013B01). Inclusion criteria were histologically confirmed prostate carcinoma and a minimum of two [^18^F]PSMA-1007 PET/CT examinations performed using the same imaging protocol. Patients undergoing PET/CT to evaluate [^177^Lu]Lu-PSMA therapy were primarily excluded. In addition, patients with incomplete clinical data or without the possibility of standardized image evaluation (e.g., post-splenectomy status) were excluded. This resulted in a final patient cohort of 99 individuals. Three groups were formed to test for potential differences in the behavior of UBU over time: Group A: evidence of UBU but no bone metastases; Group B: no focal bone uptake and no bone metastases; and Group C: evidence of bone metastases.

### 2.2. PET/CT Acquisition

All examinations were performed on a PET/CT clinical scanner (Biograph mCT^®^, Siemens Healthineers, Erlangen, Germany). PET data acquisition started 60–90 min after the intravenous application of weight-adapted 3 MBq/kg body weight (BW) [^18^F]PSMA-1007 (synthesized on a GE TRACERlab MX (GE Medical Systems, Liège, Belgium) using materials from ABX, Radeberg, Germany), and data were acquired from the skull base to the mid-thigh level over six-to-eight bed positions with an acquisition time of 2 min per bed position. PET data reconstruction was conducted using a 3D ordered subset expectation maximization algorithm (two iterations, 21 subsets, a Gaussian filter of 2.0 mm, a matrix size of 400 × 400, and a slice thickness of 2.0 mm). If no contraindications were present, a weight-adapted 90–120 mL intravenous CT contrast agent (Ultravist 370, Bayer, Leverkusen, Germany) was injected for diagnostic CT examination. PET and CT data were co-registered and evaluated using commercial software installed at our institution (syngo.via, software version 8.2.; Siemens Healthineers, Erlangen, Germany).

### 2.3. Data Analysis

Patient information such as prostate-specific antigen (PSA) levels, Gleason score, and individual antitumor therapy was obtained from in-house clinical reports. Image analysis focusing on focal bone lesions was performed according to the PROMISE criteria [19], considering the number of lesions, miPSMA score, and CT appearance. SUV_peak_, location, and CT appearance were determined for each lesion. UBU was defined as focal osseous tracer uptake above the blood pool with or without equivocal CT findings that did not meet the PROMISE criteria for osseous metastases. The diagnosis of osseous metastasis was validated for all patients based on follow-up imaging (for the initial scan) or with additional available imaging and clinical data (for the follow-up scan), in consensus with a senior nuclear medicine physician experienced in performing PET-CT evaluation (CLF).

### 2.4. Statistical Analysis

Statistical evaluation was performed using Microsoft Excel, software version 2405 (Microsoft ^®^) and R, software version 4.4.1 (open source). The data were largely of similar variance and homogeneous normal distribution. Otherwise, the Johnson correction was applied. One-way ANOVA was used to evaluate differences between groups. *p* values < 0.05 were considered statistically significant. The Bonferroni correction was used for multiple comparisons to counteract Type-1 error increases in multiple comparisons.

## 3. Results

### 3.1. Initial PET/CT Findings

A total of 99 patients (mean age 69 ± 7 years) were analyzed and divided into three groups according to initial PET/CT findings: Group A: evidence of UBU but no bone metastases, *n* = 56, with a total of 230 UBU lesions; Group B: no focal bone uptake and no bone metastases, *n* = 24; and Group C: evidence of bone metastases, *n* = 19, with a total of 28 metastases and 54 lesions rated as UBU. In total, 96/99 patients underwent local therapy (either surgery or radiation therapy), and 45/99 patients received androgen deprivation therapy, including the 3 patients without local therapy. Additionally, 10/96 patients received further treatment (either chemotherapy or secondary hormonal therapy). Detailed patient information is provided in Table 1.

There was no statistically significant difference between the three groups regarding age (*p* = 0.06), Gleason score (*p* = 0.14), injected tracer dose (*p* = 0.80), uptake time (*p* = 0.29), UBU SUV_peak_ (*p* = 0.12), and SUV_mean_ uptake in the spleen (*p* = 0.10) and parotid gland (*p* = 0.09). The SUV_mean_ of the blood pool was lower in both patients with UBU (Group A: 2.1 ± 0.6) and patients with bone metastases (Group C: 2.2 ± 0.7) compared to patients without focal bone uptake (Group B: 2.6 ± 0.5). A statistically significant difference was only found between patients with UBU and patients without focal bone uptake (*p* = 0.003). Serum PSA levels were higher in patients with UBU (3.9 ± 8.2 ng/mL) and patients with bone metastases (5.4 ± 6.6 ng/mL) than in patients without focal bone uptake (1.6 ± 1.8 ng/mL). However, a statistically significant difference was only found between patients with bone metastases and patients without focal bone uptake (*p* = 0.03).

**Figure 1:**
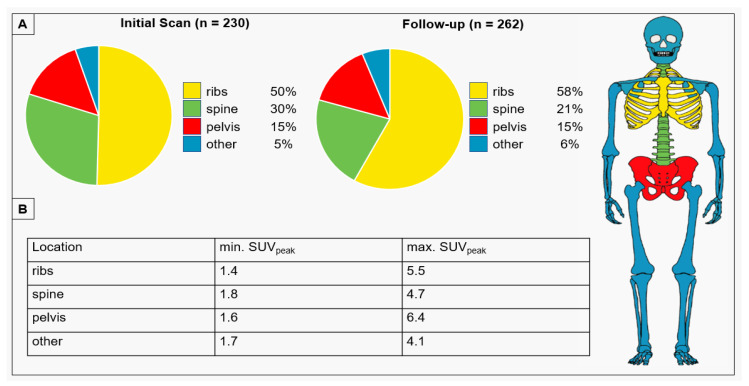
The distribution of focal unspecific bone uptake across different skeletal regions (**A**) and peak standard uptake values (SUV_peak_) across these locations (**B**).

In Group A, 56 patients had 230 UBU in the initial scan (IS). The mean SUV_peak_ of these lesions was 2.7 ± 0.8. The most common location in both cases was the thoracic rib cage, accounting for 50%. Figure 1 illustrates the distribution of UBU locations, including the IS and the follow-up examination (FU).

### 3.2. Follow-Up Examination

The FU took place 13.0 ± 7.2 months after the IS. There was no difference in follow-up interval between the groups (*p* = 0.94). In Group A (patients with UBU), 49/56 patients presented with 262 UBU but no presence of bone metastasis in the FU. The mean SUV_peak_ of these lesions (2.6 ± 0.7) was comparable to that of the IS. There was no change in miPSMA-score and CT appearance in 44/49 patients. Of these, 22/44 patients received only local treatment or no treatment between IS and FU. A total of 12/44 patients had no change in systemic treatment (androgen deprivation therapy or secondary hormonal therapy), while 10/44 patients experienced a change in or the start of systemic treatment between IS and FU.

In 5/49 patients, a change in the miPSMA score (decrease in three cases) or a change in CT appearance (change from no correlate to equivocal CT correlate in two cases) was found, but this did not lead to a change in the lesion assessment for malignancy. One of these patients was still on androgen deprivation therapy prior to IS. The others (four) had no systemic treatment between IS andFU.

New bone metastases were detected in 7/56 patients in Group A. Of these patients, six had metastases in the ribs, four had metastases in the spine, two had metastases in the pelvis, and three had metastases in other bone regions. Patient-specific analysis showed that four patients had new bone metastases, while in three patients, at least one metastasis was initially classified as UBU. The three patients in whom UBU was identified as an early bone metastasis did show the following characteristics in the first scan: Patient 1 had UBU in the ribs on the first scan with an SUV_peak_ of 2.9, which was identified as metastasis in the follow-up with an SUV_peak_ of 3.6, as well as a newly formed metastasis in the pelvis. Patient 2 showed UBU in the pelvis with an SUV_peak_ of 4.9, which was identified as metastasis in the follow-up with an SUV_peak_ of 7.1 and a suspicious CT appearance. There were also several new metastases formed in the ribs, spine, and other bone regions. Patient 3 showed UBU in the ribs, the spine, and the femur with SUV_peak_ values of 4.2, 3.3, and 7.6, respectively. UBU levels in the ribs and the spine were rated as metastases in the FU (SUV_peak_ 16.1 and 17.1), while the femoral lesion showed no increase in tracer uptake (initial SUV_peak_ of 7.6 decreased to 3.9).

**Figure 2:**
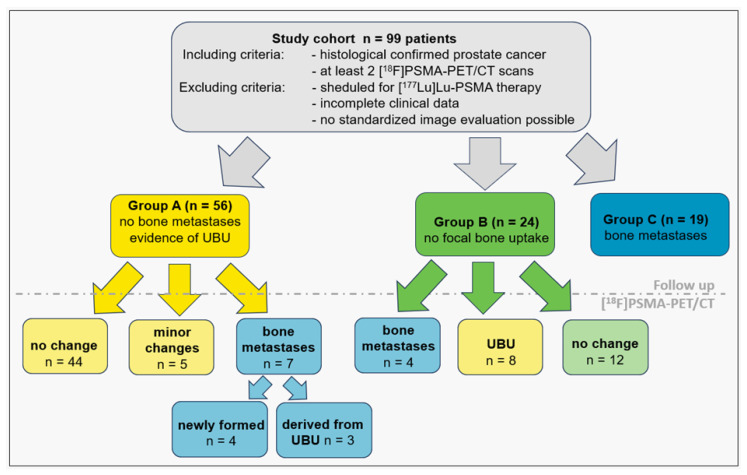
Participant flow diagram demonstrating identification and further selection of study cohort and subgroups. UBU = focal unspecific bone uptake.

In Group B (no focal bone uptake on IS), 4/24 patients (17%) developed osseous metastases on FU (*n* = 9). Figure 2 displays patient selection and follow-up evaluation.

**Figure 3:**
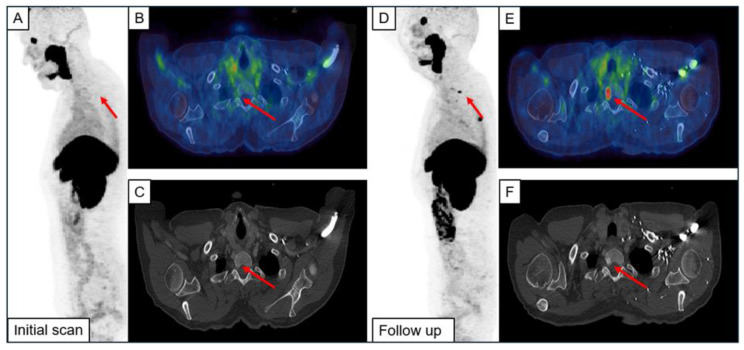

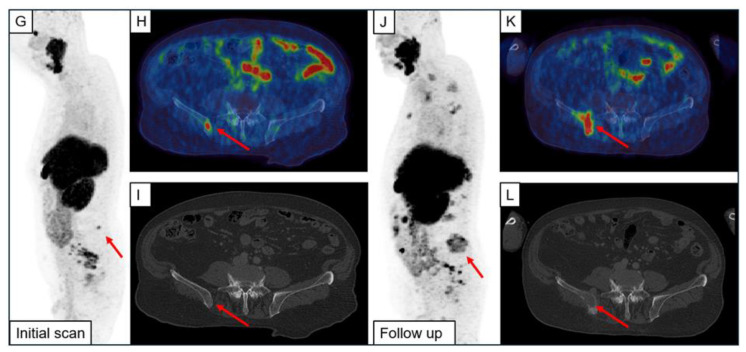
Example of a newly emerged bone metastasis (**A**–**F**) and metastases originating from focal unspecific bone uptake (**G**–**L**). The initial scan (IS) and the follow-up imaging (FU) are shown. Red arrows indicate the metastases.

An example of a newly formed metastasis and one originating from UBU is illustrated in Figure 3.

## 4. Discussion

Our study further confirms the described high prevalence of UBU in [^18^F]PSMA-1007 PET/CT. UBU without prevalence of bone metastases was most commonly located in the thoracic rib cage (50%), followed by the spine (30%). The prevalence of UBU, determined in our study to be 56%, is at the upper end of the previously reported rates for [^18^F]PSMA-1007, which were 34.2% [20], 38% [29], 41% [31], 43.9% [21], and 51.4% [15]. This variation may be attributed to differences in the detection accuracy of different PET systems, in particular the increased detection rate with digital PET scanners [15]. Furthermore, follow-up imaging of all patients using [^18^F]PSMA-1007 PET/CT revealed the malignant transformation of UBU in three patients only.

In our cohort, the PSA levels were elevated in both subgroups of patients with UBU and osseous metastases. While an increase in PSA levels upon the detection of bone metastases is highly explicable, a relationship between UBU and serum PSA level seems less obvious, especially when considering our and other pre-existing findings stating that the overwhelming majority of UBU is not associated with prostate cancer. In addition, several other studies [15,21] have failed to demonstrate an elevated PSA level in UBU, suggesting that the cause of this observation in our data remains unclear.

The current study did not identify a notable correlation between the frequency of UBU and other clinical and imaging variables, such as age, Gleason score, injected tracer dose, uptake time, or mean uptake in the spleen and parotid gland. These findings align with those of previous studies [15,21], although one study showed an increased prevalence of UBU with a 90 min uptake time compared to a 60 min uptake time [15]. In addition, a lower blood pool activity was observed in our cohort of patients with UBU. This finding can be attributed to the definition of UBU itself, as it was defined according to the PROMISE criteria, which include all lesions above the blood pool. Consequently, the dependence of UBU on particularly low blood pool activity is to be expected. A comparison with other studies regarding the SUV is constrained because it is not yet clear which is the most appropriate parameter, leading to different values, such as SUV_mean_ and SUV_peak_, but primarily SUV_max_, being used and assessed. Due to the point-spread function reconstruction utilized at our center and the resulting distortion of SUV_max_, we do not favor this parameter [32].

Our findings are consistent with the existing literature regarding UBU location. In our study, most (50%) was found in the ribs, which is comparable to the range of 57–62% reported in the other studies [15,21,29]. This is followed (to a lesser extent, depending on the specific division of the skeletal system in the study) by lesions in the pelvis or spine. This spread pattern is particularly interesting because, based on the distribution of metastases in bone scans, it has been shown that rib metastases without concurrent spinal metastases are extremely rare, occurring in only 1% of cases [33]. However, it should be noted that PSMA-PET/CT has a higher sensitivity compared to bone scans, so these results are not necessarily applicable. Biopsy studies also show that rib metastases account for only 12–20% of all osseous metastases in prostate cancer [34,35].

The prevalence of osseous metastases derived from UBU varies considerably in the literature due to variations in the definition of UBU, different radiotracers, variant scanner technology, and the methods for confirming the presence of osseous metastasis (imaging, clinical assessment, or biopsy). In a recent study with [^18^F]PSMA-1007, Luo et al. [31] reported a prevalence of focal bone uptake not classified as metastasis of only 11.6% and the presence of malignancy in 22.8% of cases when PSMA-RADS 3B (equivocal uptake in bone lesions, not defined but also not atypical of prostate cancer in anatomical imaging) [36] was used, which nevertheless exhibited a significantly lower prevalence than those reported in other studies [15,20,21,29].

Grünig et al. demonstrated a higher proportion of malignancy (14%) in cases with detected UBU. Of note, follow-up data were available for only 36% of patients and were inconsistent, sometimes relying solely on conventional imaging [15].

Furthermore, Arnfield et al. found no evidence of malignant transformation among patients with UBU (UBU detection rate 43.9%) [21]. However, follow-up data were available for only 82% of the patients, of whom only 36% underwent follow-up imaging.

When placing our findings in the context of the existing literature, we would like to emphasize that although our study lacked histological verification and entailed a degree of uncertainty, we utilized [^18^F]PSMA-1007 PET/CT imaging for the entire cohort, recognized as the optimal modality for patient monitoring.

Through this approach, we identified the malignant transformation of UBU in 5% of cases at the patient level, corresponding to 4% of initially non-osseous metastatic patients in our cohort (*n* = 80; Groups A and B). Nevertheless, it is imperative to acknowledge the persistent risk of new metastatic occurrences in the presence of an underlying malignancy. We showed that the risk of new osseous metastases was 10% (based on all patients initially classified as non-osseous metastatic). This risk appears to be independent of the presence of UBU and surpasses that of UBU transformation to metastasis nearly threefold.

Regarding risk factors that could indicate the risk of a malignant origin of UBU, Luo et al. proposed positive lymph node metastasis, an elevated SUV_max_, and pelvic localization [31]. Arnfield et al. suggested a threshold of SUV_max_ 7.2 to classify lesions as likely benign [21]. Due to the presence of only three patients with UBU-derived bone metastases in our cohort, we cannot conduct a statistical analysis on this matter or other risk factors. However, given the low individual SUV values of these lesions (2.9, 3.3, 4.2, and 4.9), we can at least not support the hypothesis of SUV dependence.

The limitations of our study include the limited number of patients and the retrospective design. In addition, the uptake of our lesions tended to be in the lower range (where comparable between different SUV parameters), thus only allowing limited conclusions regarding UBU with high uptake, yet not classified as metastasis according to the PROMISE criteria. However, as our data showed, the number of such lesions is limited in everyday practice. Furthermore, the fact that the scans were performed with different clinical indications could introduce bias but can be considered legitimate given the incidental nature of UBU. The clear differentiation from early osseous metastases, especially in cohorts with low prevalence of osseous metastases, such as patients with biochemical recurrence after prostatectomy, is challenging. It is likely that the very high prevalence of UBU may have a significant impact on the management of several patients. This influence may range from potential under- or over-treatment in the worst-case scenario to more commonly additional or more frequent imaging investigations. In any case, uncertainty remains for treating physicians and patients, even if there is no direct impact on therapeutic decisions.

## 5. Conclusions

Our study underscores the lack of significant differences between patients with or without UBU concerning various imaging and clinical parameters. Furthermore, only a small fraction of the detected UBU turned out to be metastases over time (2%). This proportion is lower than the likelihood of developing new osseous metastases, which also appears to be independent of the presence of UBU. Therefore, our data suggest that the presence of UBU, analogous to the PROMISE criteria, does not influence the development of osseous metastases over time. Based on this data, treatment decision-making should not be significantly influenced by the findings of UBU.

## Figures and Tables

**Table 1:** Patient characteristics.

	Group A (*n* = 56)	Group B (*n* = 24)	Group C (*n* = 19)
Age [years]	70 ± 6	66 ± 9	69 ± 7
Gleason score: 6–7 [*n*] 8–10 [*n*]	35 21	11 13	10 9
Initial serum PSA [ng/mL]	3.9 ± 8.2	1.6 ± 1.8	5.4 ± 6.6
Tracer dose [MBq]	317 ± 17	314 ± 13	314 ± 12
Uptake time [min]	72 ± 14	66 ± 12	71 ± 14
SUV_mean_ blood pool	2.1 ± 0.6	2.6 ± 0.5	2.2 ± 0.7
SUV_mean_ spleen	10.8 ± 3.9	8.9 ± 2.1	10.8 ± 4.9
SUV_mean_ parotid gland	17.4 ± 4.9	18.6 ± 4.7	20.3 ± 5.1
UBU [*n*]	230	0	54
UBU SUV_peak_	2.7 ± 0.8	-	2.6 ± 0.5
Metastases [*n*]	-	-	28
Metastases SUV_peak_	-	-	7.7 ± 8.1

PSA = prostate-specific antigen; SUV = standard uptake value; UBU = focal unspecific bone uptake.

## Data Availability

The data presented in this study are available on request from the corresponding author.
